# The Effect of the Modification of Mica by High-Temperature Mechanochemistry on the Anticorrosion Performance of Epoxy Coatings

**DOI:** 10.3390/polym13030378

**Published:** 2021-01-26

**Authors:** Yahui Cai, Fandi Meng, Li Liu, Rui Liu, Yu Cui, Hongpeng Zheng, Fuhui Wang

**Affiliations:** 1 Shenyang National Laboratory for Materials Science, Northeastern University, Wenhua Rd 3-11, Shenyang 110819, China; 1870279@stu.neu.edu.cn (Y.C.); Zhenghongpeng@mail.neu.edu.cn (H.Z.); fhwang@mail.neu.edu.cn (F.W.); 2 Shenyang National Laboratory for Materials Science, Institute of Metal Research, Chinese Academy of Sciences, Wencui Road 62, Shenyang 110016, China; rliu15s@imr.ac.cn; 3 Shi-Changxu Innovation Center for Advanced Materials, Institute of Metal Research, Chinese Academy of Sciences, Wencui Road 62, Shenyang 110016, China; ycui@imr.ac.cn

**Keywords:** epoxy coating, mica, high-temperature mechanochemistry, corrosion resistance

## Abstract

Epoxy resin was directly grafted onto the surface of mica powder by high-temperature mechanical ball milling. This method was used to achieve a chemical reaction between the epoxy resin and mica that cannot be carried out under conventional circumstances. The results show that an epoxy resin layer with a thickness of approximately 10 nm formed on the surface of the mica. This modified mica filler exhibited a significant change in its hydrophilic properties. The dispersion of mica and its compatibility with organic coatings also significantly improved. In addition, the modified mica filler was added to the epoxy coating. The improvement of the coating’s compactness and toughness is the reason for its anti-corrosion performance enhancement.

## 1. Introduction

Epoxy resin has excellent adhesion; low shrinkage; and good permeability resistance to water, acids, alkalis, and other corrosive media. Thus, it has been commonly used as an anti-corrosion coating in both industrial and daily-life applications [1,2,3,4,5,6,7]. Since the network structure of epoxy resin is inevitably penetrated by corrosive media, it is necessary to add various inert pigments to further improve the shielding performance of the coating, such as basalt, iron oxide, glass flakes, and so on [8,9,10,11,12]. Mica is one of the most widely used inorganic fillers in this coating [13,14,15]. As a natural sheet silicate composed of silicon ox tetrahedra and aluminium–hydroxy octahedrons, mica not only has good physical stability and anti-ageing properties but also has a two-dimensional sheet layer structure that can form a parallel arrangement in the coating. Through the maze effect, mica can theoretically improve the anti-permeability and strength of the coating [16]. In addition, it can reduce the number of high-priced pigments and decrease the costs. However, there is often an obvious interface compatibility problem between inorganic fillers and organic resins, as the inorganic fillers are hydrophilic and oil-phobic. Direct additions of inorganic mica into organic coatings tend to result in poor dispersion, agglomeration, and the formation of a large number of pores and cracks in the coating, which lead to the degradation of the coating performance [17].

Various physical and chemical methods are often used for the surface modification of fillers to solve the problem of interface compatibility between fillers and resins [18,19,20,21,22]. At present, mica modifications mainly focus on the intermediate bridging method of the coupling agent. For example, Yun et al. [23] modified the surface of mica through TiO_2_ adsorption on mica in an acidic environment, which improved the whiteness and sun protection factor of mica. Hu et al. [24] used a chemical method to graft poly (ethylene glycol)-maleic anhydride-acrylic acid (PEG-MA-AA) onto the surface of mica, thereby facilitating the dispersion performance of mica. Although these methods have enhanced the interface compatibility between the mica and the organic coating to some extent, the technology of the intermediate coupling agent is too complex to industrialize. Besides, the strength of physical grafting is weak, and the coating tends to easily detach from the substrate [25,26,27]. Therefore, finding a simple and efficient modification method with a high graft rate and graft strength has become a hot research topic.

The mechanochemical method is widely utilized to increase the reactivity of reactants by accumulating mechanical energy through grinding, compression, and other actions, causing changes in the physical and chemical properties and even the structures of materials [28,29,30,31]. The mechanochemical method is often used to induce solid reactions that are unfavorable under normal conditions and has been widely studied and applied because of its advantages of high efficiency, environmental protection, and low energy requirements. However, there are few reports on the application of mechanochemistry in the field of coating fillers. Whether it can be used for modification is unclear.

To answer this question, traditional mechanical ball milling was first used to try to graft epoxy resin onto the surface of mica, but this could not be successfully achieved. To this end, high-temperature mechanochemistry was tested in the hope of further increasing the energy input by providing thermal energy so that the mica and epoxy resin organic molecules would bond to complete the modification of the surface of the mica. In this article, the feasibility of the high-temperature mechanochemical modification of inorganic fillers was first studied, and organic epoxy resin was successfully grafted onto the surface of mica. High-temperature mechanochemical modification simplifies the modification process, increases the interface compatibility between the modified mica and the epoxy coating matrix, and thus improves the corrosion resistance of the coating. The main process is to open the epoxy-functional group in the epoxy resin through high temperatures and mechanochemistry to make it react with the hydroxyl group on the surface of the mica, thereby achieving the surface modification of the mica. The methods used in this paper—Fourier transform infrared spectrometry (FTIR), thermogravimetric analysis (TGA), and transmission electron microscopy (TEM)—indicated that the graft modification of organic epoxy molecules on the surface of mica was successfully achieved. At the same time, a corrosion test showed that the corrosion resistance of the modified mica epoxy coating was greatly improved, and the mechanism of the modified mica’s effect on the coating performance improvement was discussed.

## 2. Materials and Methods

### 2.1. Materials

Commercially available mica powder of 25 μm size (the average particle size) was used (Henan Bogrun New Material Technology Co., LTD., Henan, China). An epoxy resin-based coating was used in this study and consisted of an E44 epoxy resin (bisphenol A, Wuxi Resin Factory, Wuxi, China) as the binder, polyamide (TY-650, Tianjin Yanhai Chemical Co., Ltd., Tianjin, China) as the curing agent, and xylene as the solvent, with a mass ratio of 1:0.8:0.4 for a stoichiometric reaction.

### 2.2. Preparation of Modified Mica

The epoxy resin and mica were weighed and mixed according to a mass ratio of 1:1, and the mixture was added into the grinding tank of the 500 mL high-temperature ball mill for the grinding test. Meanwhile, agate balls with the material ratio of 5:1 were used as the grinding medium. The grinding time was 6 h, the rotation speed was 600 r/min, and the experimental temperatures were set to 25 °C and 200 °C. At the end of the experiment, the reacted mixture was repeatedly filtered and washed at least three times with a mixed solvent (xylene and n-butanol in a ratio of 7:3) to remove the unreacted epoxy resin from the mica surface. The modified product was then dried in a 60 °C oven for 24 h.

### 2.3. Testing and Characterization

The modified mica powder was dispersed in a mixed solvent and sonicated for 10 min to make a suspension, and the particle size distribution of the modified mica was measured with an ultrasonic particle size analyzer (Master sizer 2000). A sedimentation bottle was used for the sedimentation experiment. A thermogravimetric analyzer (Mettler Toledo Instruments, Shanghai, China) was used to analyze the degradation process of the organic resin on the surface of the modified mica during the heating process with a nitrogen atmosphere. Transmission electron microscopy (JEOL, JEM-2100F) was used to observe the microstructure of the mica powder surface. A Fourier transform infrared spectrometer (VERTEX70, German Bruker Instruments, Karlsruhe, Germany) was used to observe the epoxy functional groups on the mica surface (spectral range of 500–4000 cm^−1^). A contact angle tester (German Kruss DSA25, Hamburg, Germany) was used to test the wettability of the mica surface; the droplet nature in the measurements was a solution formed by dissolving epoxy resin in a mixed solvent (xylene and n-butanol in a ratio of 7:3).

The coating samples were immersed in 3.5 wt.% NaCl solution to carry out electrochemical impedance spectroscopy (EIS, Princeton P4000A Electrochemical workstation-AMETEK Trading (Shanghai) Co., Ltd., Shanghai, China) tests. The working area was 4 cm^2^. A scanning electron microscope (SEM, JEOL, JSM-7001F) was used to observe the microscopic morphology of the cross-section of the coating sample. A salt spray test chamber (Shanghai Linpin Instrument Stock Co., Ltd., Shanghai, China, LRHS-412-RJY) was used to test the salt spray resistance of the coating; five parallel samples were set for each experiment, and the temperature was set at 35 °C.

## 3. Results

### 3.1. Feasibility Study

**Figure 1:**
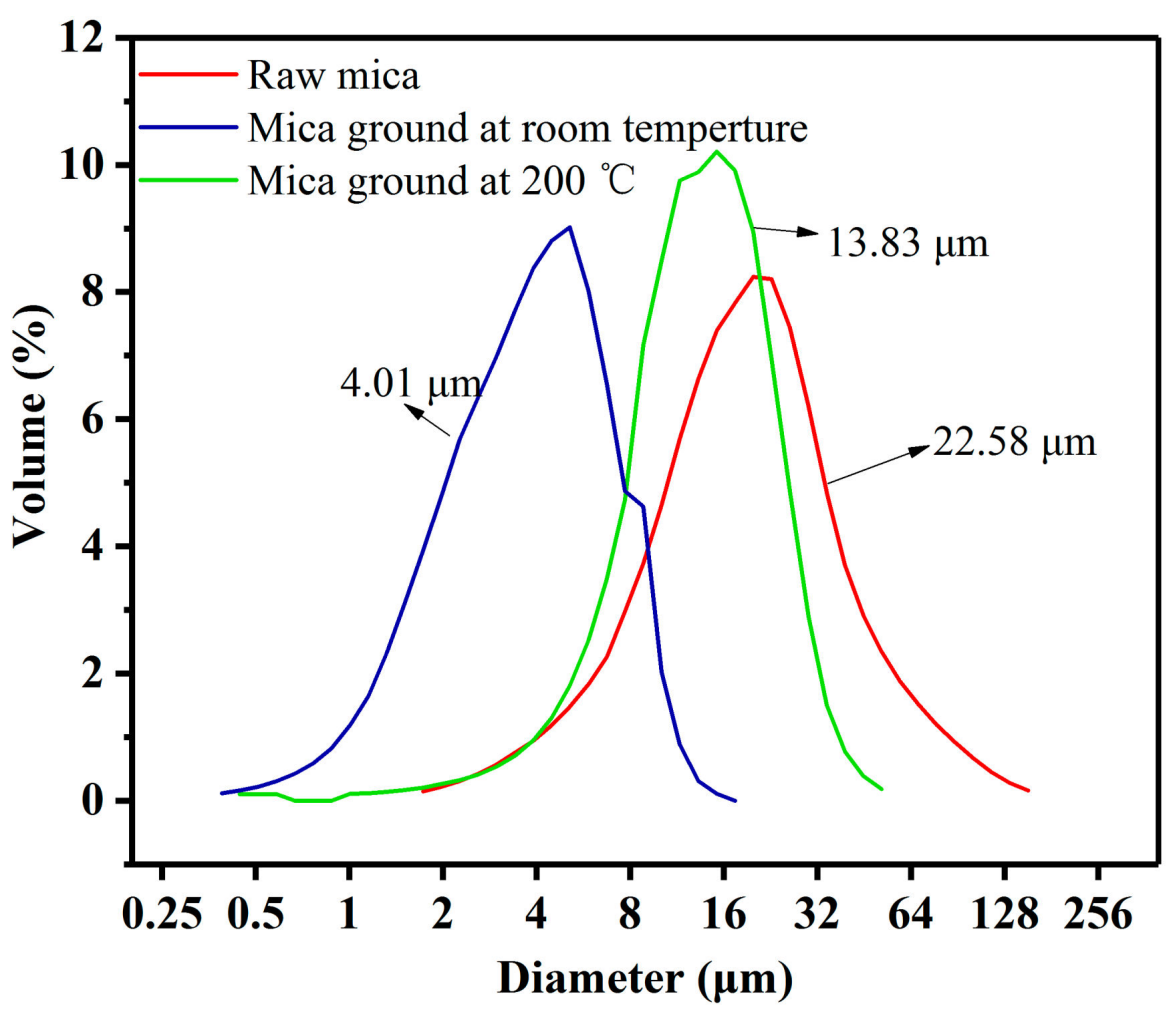
The particle size distribution and average particle size of raw mica, mica ground at room temperature, and mica ground at 200 °C.

In order to study the feasibility, experiments on unmodified mica, mechanochemically modified mica at room temperature, and mechanochemically modified mica at 200 °C were designed. The particle size distributions of the three groups are shown in Figure 1. The average particle size of the unmodified mica is approximately 22.58 μm. Under the mechanochemical modification treatment at room temperature, the average particle size of the mica drops to 4.01 μm. This is due to the continuous crushing and refinement of the mica particles under the action of the mechanical force. In contrast, the high-temperature mechanochemical treatment reduced the average particle size of the mica particles to 13.83 μm, which was mainly caused by the dual actions of high temperature and mechanical force. On the one hand, mechanical force makes the powder particles continue to break and refine; on the other hand, the high temperature makes the small particles of powder grow, so the particle size depends on the combined effects of the above two factors.

**Figure 2:**
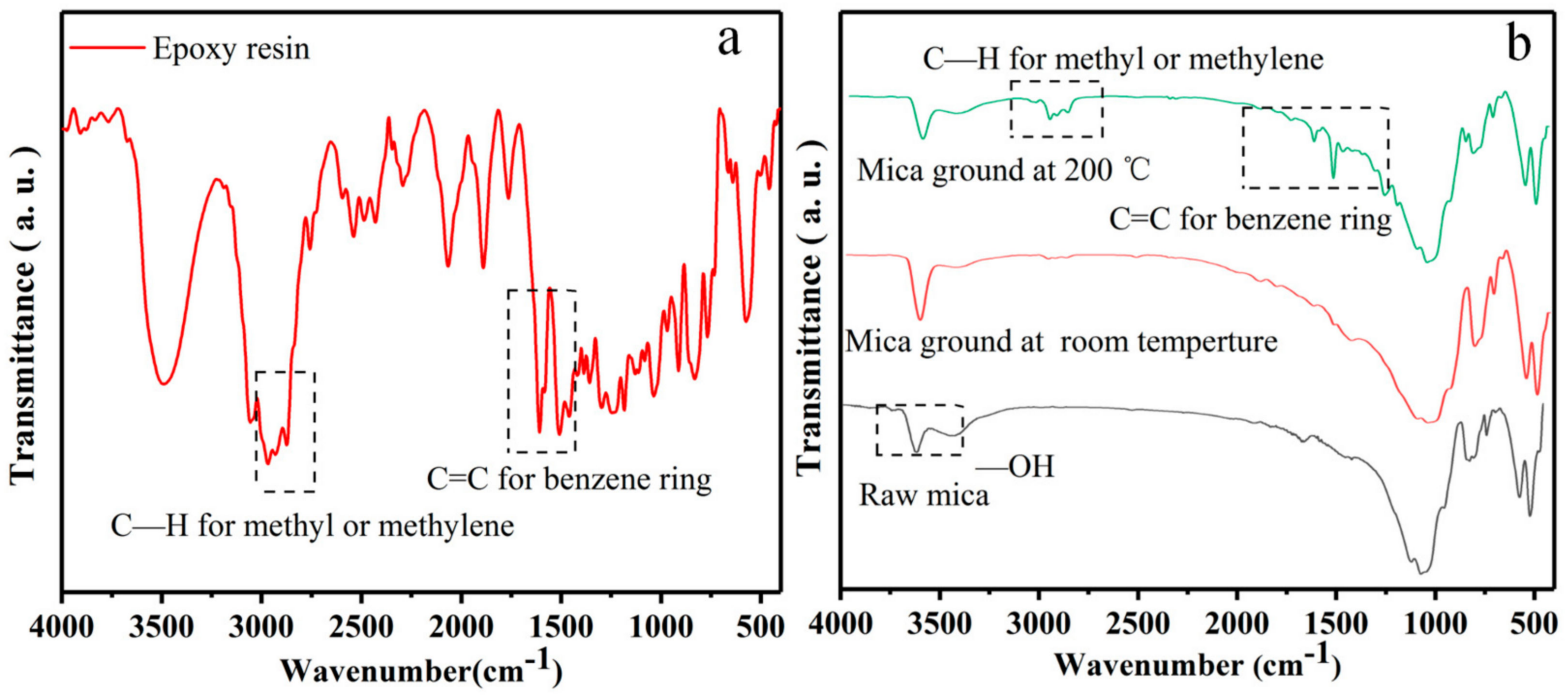
The FTIR spectrum of (**a**) epoxy resin and (**b**) mica under different conditions.

Figure 2 shows the FTIR spectrum of (a) epoxy resin and (b) mica under different conditions. As shown in Figure 2a, the FTIR spectrum of epoxy resin with three peaks at 2970 cm^−1^, 2920 cm^−1^, and 2810 cm^−1^ caused by –CH_3_ and –CH_2_ stretching vibrations, and those at 1620 cm^−1^ and 1510 cm^−1^ are benzene ring stretching vibrations [31], and the FTIR spectrum of unmodified mica (Figure 2b) with main peaks at 3620 cm^−1^ and 3420 cm^−1^ (–OH stretching vibration), 1020 cm^−1^ (Si–O–Si stretching) and 770 cm^−1^ (Si–O stretching vibration) indicates the presence of hydroxide groups and silico–oxygen bonds [32]. Compared with the FTIR spectrum of unmodified mica, the FTIR peaks of mica under the mechanochemical treatment at room temperature did not change significantly, which indicates that the surface modification of mica was not completed under those conditions. On the other hand, in the FTIR spectrum of mechanically modified mica at the high temperature of 200 °C, new peaks at 2970, 2920, and 2810 cm^−1^ were caused by –CH_3_ and –CH_2_ stretching vibrations, and those at 1620 and 1510 cm^−1^ indicate benzene ring stretching vibrations. The new peak position is close to the absorption peak position of the group in the epoxy resin molecule, which shows that high-temperature mechanochemistry has completed the graft modification of the mica surface. The above results show that high-temperature mechanochemistry can trigger a reaction to complete the surface modification of mica.

**Scheme 1:**
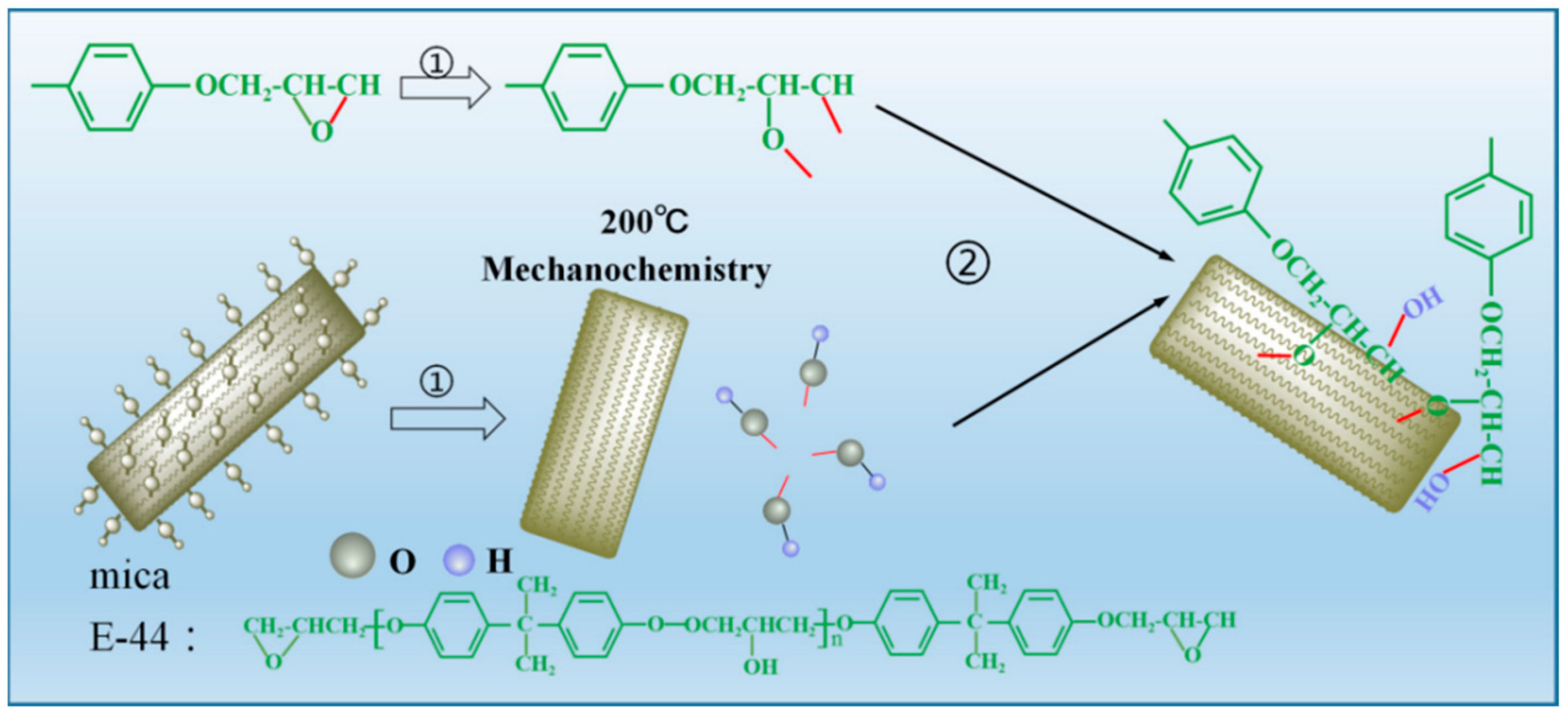
Structural diagram of the epoxy resin-grafted surface-modified mica.

For the FTIR curve of mica after high-temperature mechanochemical modification, the corresponding methyl or methylene stretching vibration of the epoxy resin and the benzene ring stretching vibration peaks appeared, which proves the presence of the organic epoxy resin on the surface of the mica. However, the modified epoxy resin did not show the original vibration peak of the epoxy group. This result indicates that there exists a bonding reaction between the epoxy resin and the hydroxyl group on the surface of the mica during the modification process. Organic resin is successfully grafted onto the surface of the mica. The specific reaction process is schematically represented in Scheme 1. The reaction process is divided into the following two steps: ① under the high-energy environment of high-temperature mechanochemistry, the epoxy bond in the epoxy and the hydroxyl bond in the mica are opened respectively, and the surface of mica and epoxy, respectively, forms high-energy active sites that can participate in the chemical reaction; ② under the continuous action of high energy environment, the broken epoxy bond of epoxy resin reacts with the surface active sites of mica to form a new covalent bond, thus completing the modification work.

**Figure 3:**
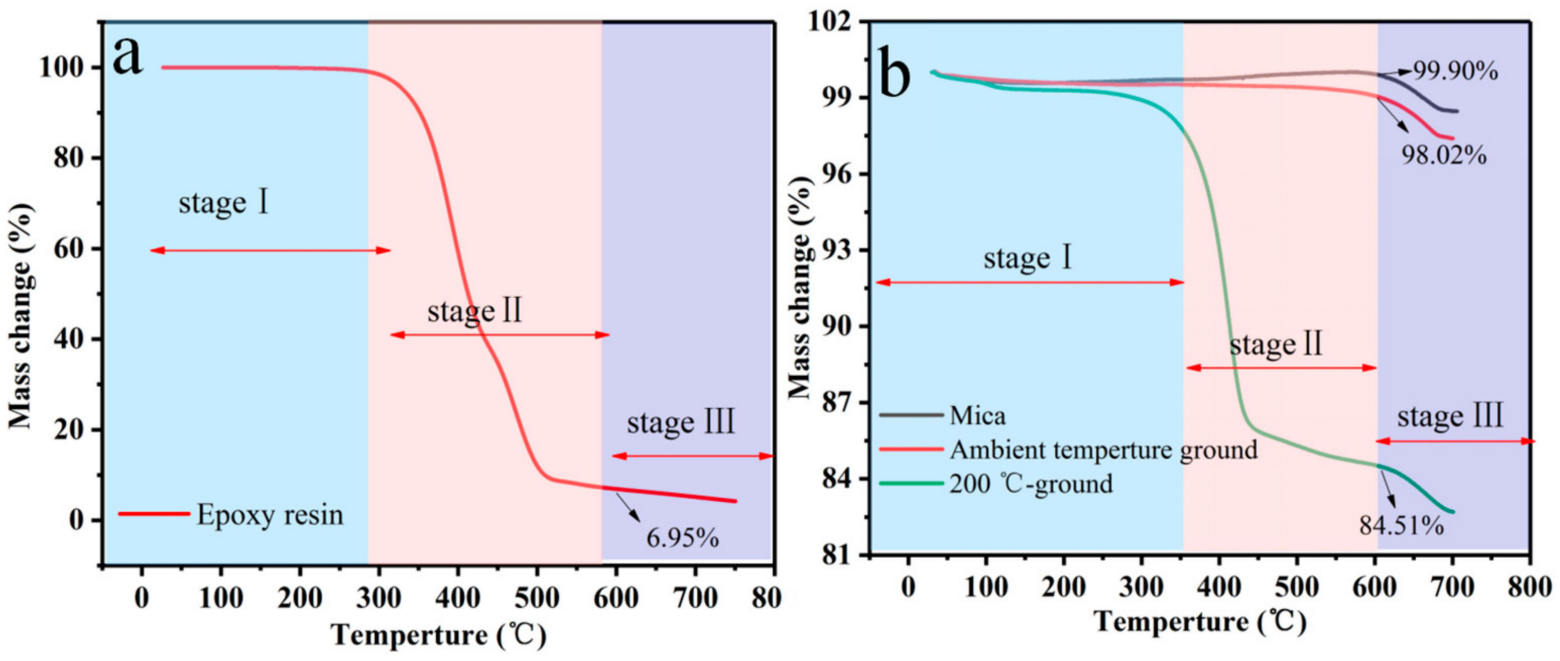
TGA curves of (**a**) epoxy resin and (**b**) raw mica, mica ground at room temperature, and mica ground at 200 °C.

Figure 3 shows the TGA weight loss curves of epoxy resin, raw mica, mica ground at room temperature, and mica ground at 200 °C. As shown in Figure 3a, the weight loss of pure epoxy mainly occurs between 300–600 °C. When the temperature rises to 600 °C, the weight loss rate of epoxy reaches 93.05%, which is mainly caused by the decomposition of organic epoxy molecules in this interval.

On the other hand, the TGA curve of the modified mica (Figure 3b) represents the amount of epoxy grafting on the surface of the mica. The greater the weight loss of the TG was, the more epoxy molecules on the surface of mica particles and the better the grafting effect. Figure 3b shows that the weight loss rate of unmodified mica at 600 °C is approximately 0.1%, and that of the mechanochemically modified mica at room temperature is approximately 1.98%. The weight-loss rates of the two are similar, both of which are caused by the volatilization of water molecules and other impurities contained in the powder particles. The weight loss rate of high-temperature mechanochemically modified mica particles was approximately 15.49%. This result is mainly due to the thermal decomposition of epoxy molecules on the mica surface during the heating process. The weight loss process can be roughly divided into the following three stages. Stage 1 is in the range of 25 to 300 °C. This stage has a low weight-loss rate that is caused by the high-temperature volatilization of water molecules and other impurities in the powder. In stage 2, in the range of 300 to 600 °C, the sample experienced significant weight loss, which was mainly caused by the thermal decomposition of the organic molecules of the epoxy resin on the surface of the mica. In stage 3, the samples continued to lose weight at 600 to 700 °C, mainly due to the loss of sample mass caused by hydroxy dehydration inside the mica as a result of the high temperature. This result shows that high-temperature mechanochemistry has completed the surface modification of mica.

### 3.2. Characterization of the Surface Properties and Morphology of Mica Particles

**Figure 4:**
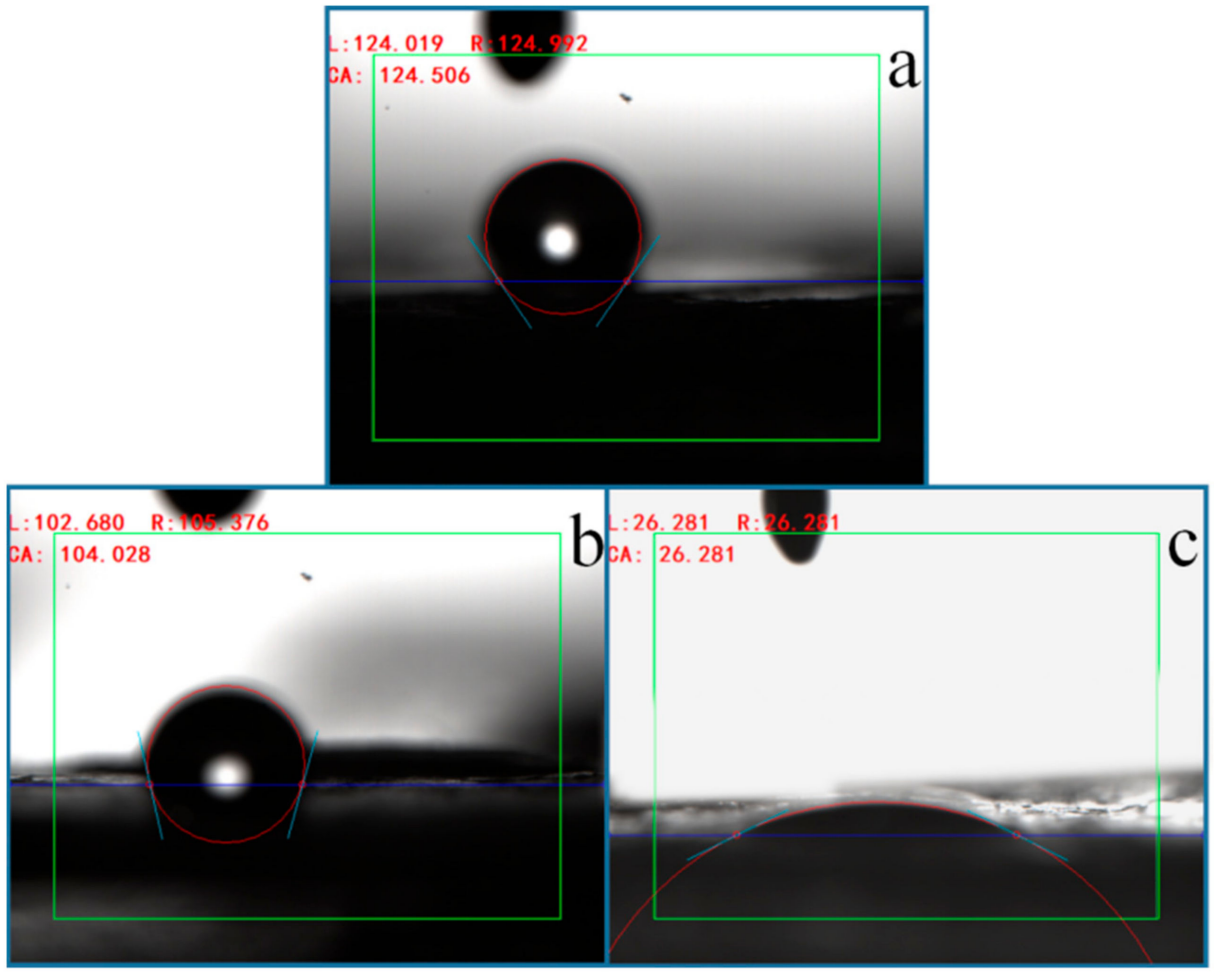
Contact angles of (**a**) raw mica, (**b**) mica ground at room temperature, and (**c**) mica ground at 200 °C.

Figure 4 shows the contact angles of (a) raw mica, (b) mica ground at room temperature, and (c) mica ground at 200 °C. The droplet nature in the measurements is a solution formed by dissolving the epoxy resin in a mixed solvent (xylene and n-butanol in a ratio of 7:3). As shown in Figure 4, The contact angles of the unmodified mica and mechanochemically modified mica at room temperature were 124.5 and 104.0, respectively, and the contact angle of mica did not change obviously after modification. On the other hand, the contact angle of the mica particles after high-temperature mechanochemical modification decreased to 26.3. This indicates that the lipophilic properties of modified mica were significantly improved. Combined with the FTIR results, this was caused bythe lipophilic epoxy resin covering the surface of the mica particles, which changed the surface properties of the mica and greatly improves the lipophilic properties of the mica.

**Figure 5:**
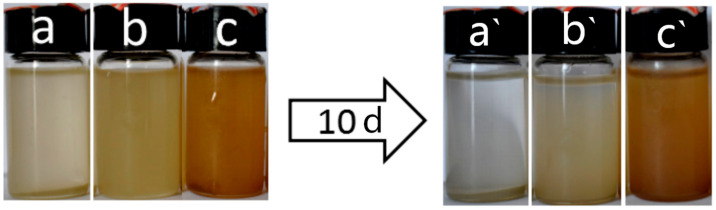
Digital photographs of the sedimentation of (**a**) raw mica, (**b**) mica ground at room temperature, and (**c**) mica ground at 200 °C.

Photographs of the sedimentation for different samples are presented in Figure 5. The sedimentation experiment shows the interface compatibility of mica particles with epoxy resin. After 10 days, both the unmodified and the room-temperature mechanochemically modified samples showed obvious sedimentation, while the high-temperature mechanochemically modified samples showed a certain degree of settlement, but it was not obvious. Coupled with the above contact angle test results, the presence of the organic resin on the surface of mica changes the hydrophilicity of mica and ultimately improves the compatibility of mica particles in epoxy resin.

**Figure 6:**
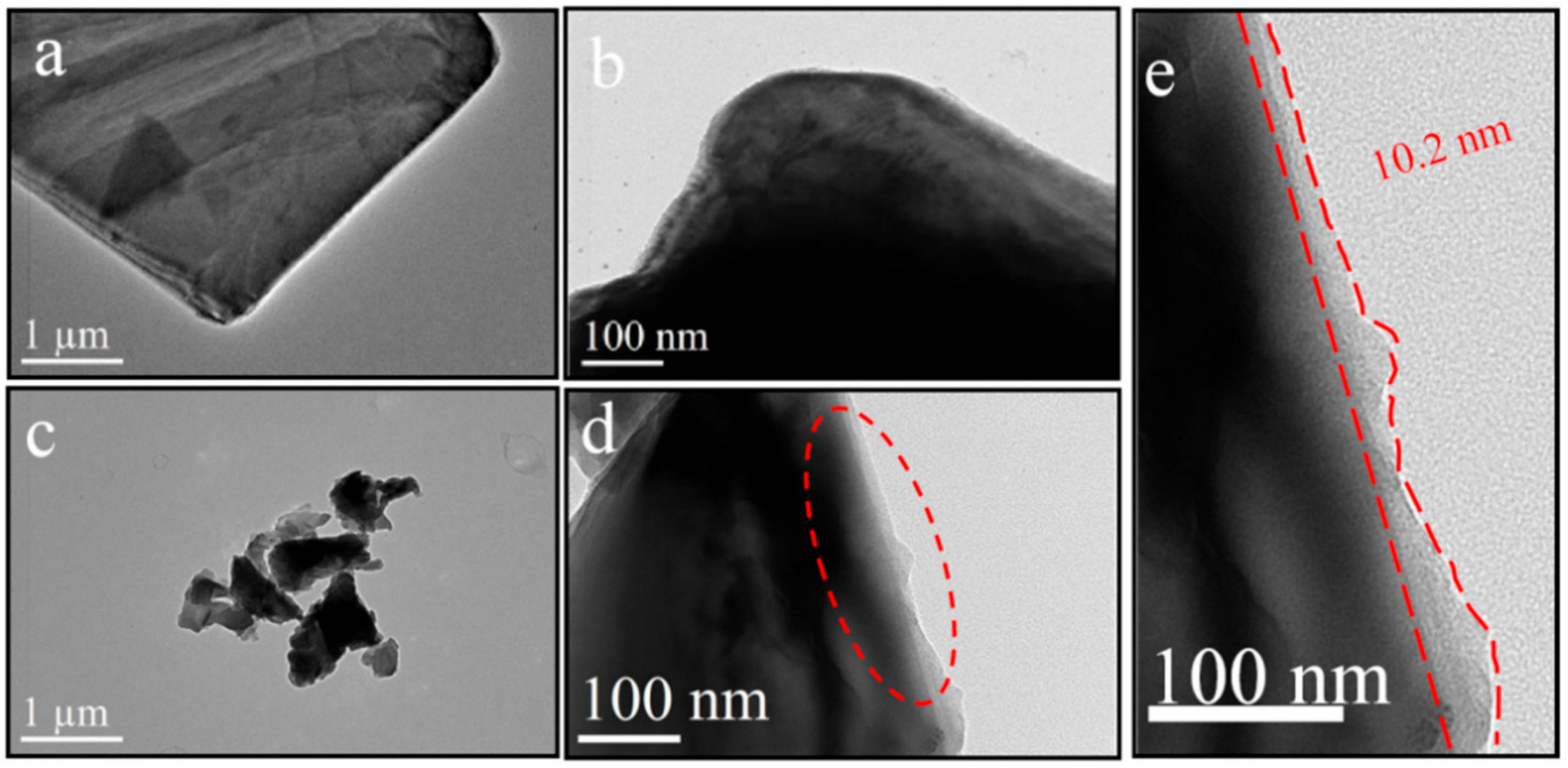
TEM images of (**a**,**b**) unmodified mica, and (**c**–**e**) modified mica.

Figure 6a–e show TEM images of mica particles before and after modification, respectively. Compared with unmodified mica, a thin layer appears on the surface of the high-temperature mechanochemically modified mica. Based on the analysis above, this film is an organic molecular layer of epoxy resin; the enlarged figure shows that the thickness of the epoxy resin layer on the mica surface is approximately 10 nm. Importantly, its presence reduces the surface activity of the mica, prevents further agglomeration between particles, changes the wettability of the mica surface, increases the contact angle, and effectively improves the compatibility between the mica and the epoxy resin.

### 3.3. Characterization of Coating Performance

**Figure 7:**
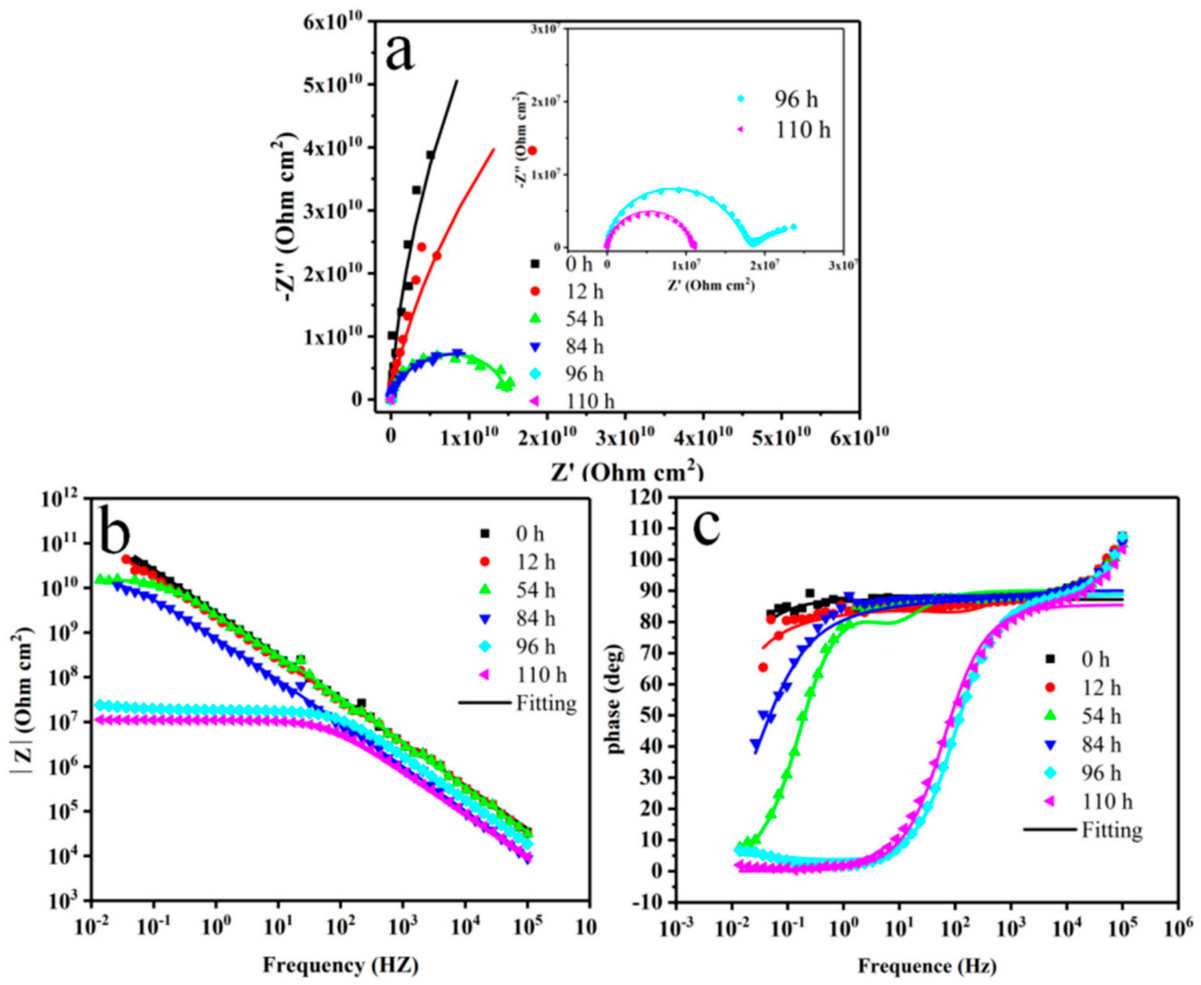
Impedance plots (**a**–**c**) of neat epoxy resin coatings immersed in a 3.5 wt.% NaCl solution.

N EIS analysis of the neat epoxy resin coatings obtained during exposure to a 3.5% NaCl solution, Bode plots, and Nyquist plots are shown in Figure 7 for different exposure times. In general, the impedance modulus at a low frequency (|Z|_0.01Hz_) is used to characterize the corrosion protection property of the coating [33]. As shown in Figure 7, at the initial stage of immersion, the epoxy coating had better corrosion resistance. The coating presented a larger semicircle (Figure 7a)—|Z|_0.01Hz_ was 5.11 × 10^10^ Ω cm^2^ (Figure 7b)—the frequency range close to 90°was reduced to between 10^−1^ and 10^5^ Hz (Figure 7c). As the immersion time increased, the coating quickly failed. After 110 h, the performance of the coating dropped significantly, presenting a smaller semicircle—|Z|_0.01Hz_ dropped to 1.79 × 10^7^ Ω cm^2^—and the frequency range (Figure 7c) close to 90° was reduced to between 10^3^ and 10^5^ Hz. The main reason for the decline in the anticorrosive performance of epoxy coatings is that the coating itself has a large number of unavoidable holes and defects. Water molecules diffuse rapidly through these holes and defects, making the coating quickly fail in a short time. Therefore, it is necessary to add inorganic fillers to the epoxy coating.

**Figure 8:**
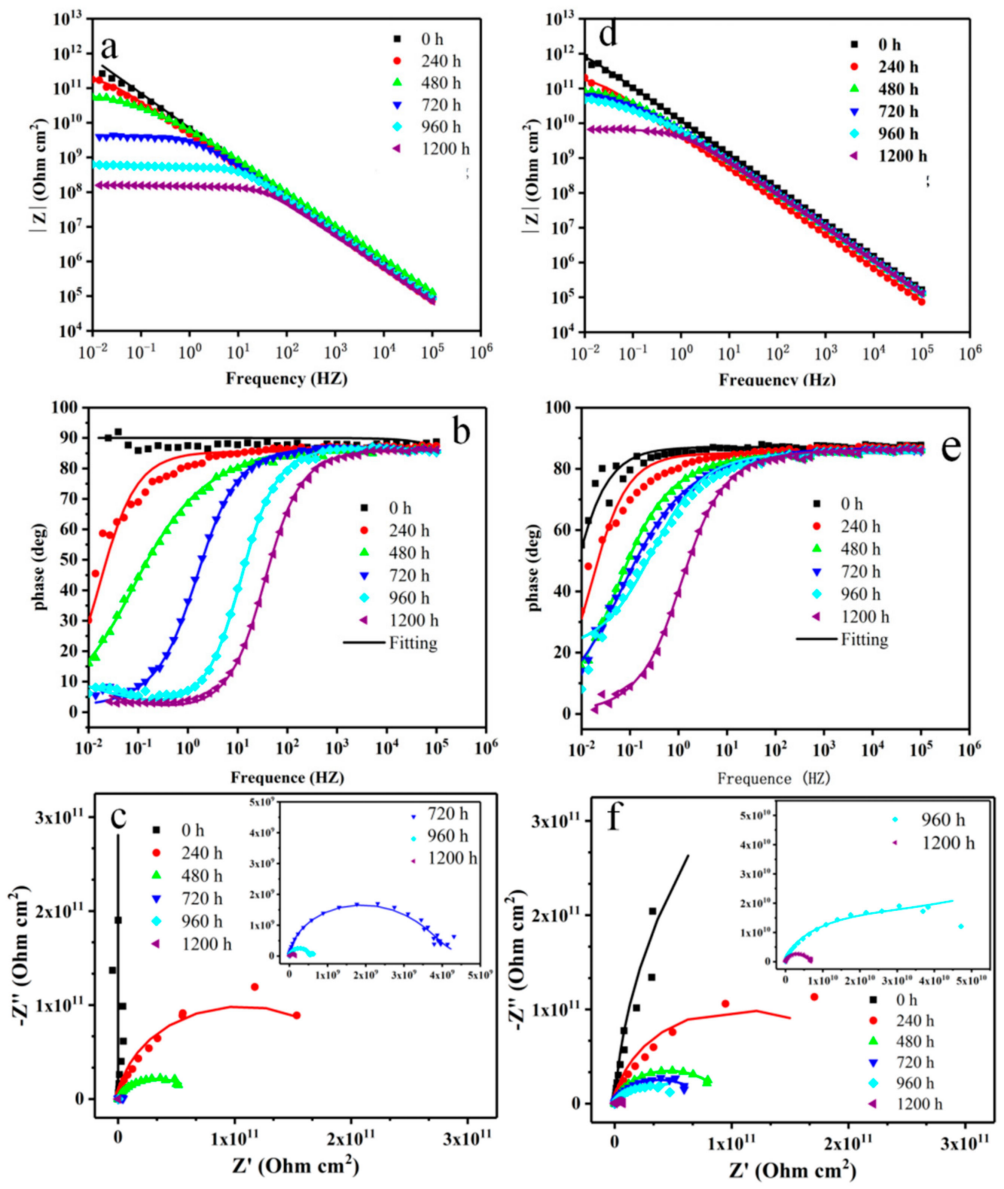
Impedance plots of (**a**–**c**) unmodified and (**d**–**f**) modified mica coatings immersed in 3.5 wt.% NaCl solution.

Figure 8 shows impedance plots of (a–c) unmodified and (d–f) modified mica coatings immersed in a 3.5 wt.% NaCl solution Initially, the unmodified mica coating had a |Z|_0.01Hz_ value of 4.5 × 10^11^ Ω cm^2^ (Figure 8a). After 240 h of immersion, the |Z|_0.01Hz_ reduced to 1.8 × 10^11^ Ω cm^2^. After 1200 h of immersion, the |Z|_0.01Hz_ decreased to 1.6 × 10^8^ Ω cm^2^. With an increased immersion time, the low-frequency impedance decreased continuously. This indicates that the unmodified mica coating is not durable upon long-term immersion in a 3.5% NaCl solution. For the modified mica coating (Figure 8d), the initial |Z|_0.01Hz_ value was about 7.8 × 10^11^ Ω cm^2^ and attained the highest value among all the samples. After 240 h of immersion, it was still at 1.7 × 10^11^ Ω cm^2^. The |Z|_0.01Hz_ gradually decreased to 5.0 × 10^10^ Ω cm^2^ after 960 h of immersion and maintained a high corrosion resistance (6.7 × 10^9^ Ω cm^2^) until 1200 h of immersion. It can be seen that the |Z|_0.01Hz_ value of the modified mica coating was one and two orders of magnitude higher than that of the unmodified mica coating. The modified coatings provided better corrosion protection than the unmodified coatings with the same content.

At the same time, in the initial stage of immersion, the frequency range of the two coating phase angles close to 90° was 10^−1^ to 10^5^ Hz, as shown in Figure 8b,e. With an increased immersion time, the frequency range of the two coatings gradually reduced. After 1200 h of immersion, the frequency range of the unmodified mica coating was reduced to between 10^3^ and 10^5^. By contrast, the frequency range of the modified coating phase angles close to 90° was wider than that of the unmodified mica coating, which was 10^0^ to 10^5^. In general, the larger the semicircle diameter in the Nyquist graph, the better the corrosion resistance of the coating [33]. It can be observed from the Nyquist diagrams in Figure 8c,f that the modified mica coating presents a larger semicircle during the entire immersion process. This indicates that the modified mica coating has better corrosion resistance.

**Figure 9:**
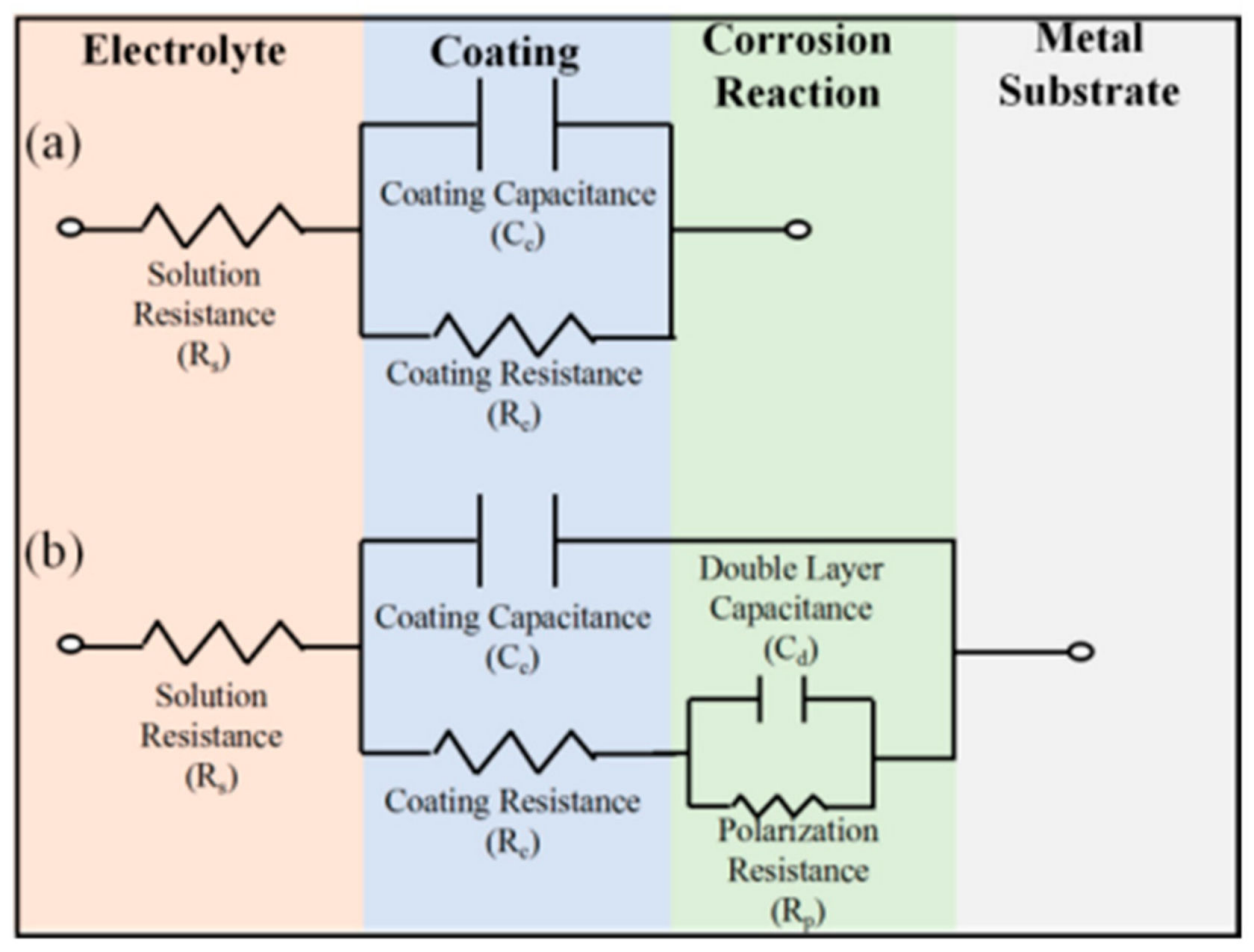
Equivalent electrical circuit of coatings (**a**,**b**).

Figure 9 shows the corresponding equivalent electrical circuit for fitting the EIS data. As shown in Figure 9, Rs is the solution resistance, which is the resistance between the working electrode and the reference electrode, Cc and Rc represent the coating capacitance and coating pore resistance, respectively, Cd is the constant phase element representing the double layer capacitance, and Rp represents the polarization resistance. In the initial stage (0–240 h) of immersion, Nyquist plots of the unmodified and modified mica coatings both exhibit a characteristic of a pure capacitive loop, and the electrical equivalent circuit in Figure 9a is used for fitting the procedure. With the increased immersion time (480–1200 h), the second capacitive loop appeared in the Nyquist plots for the modified and unmodified coatings, and the experimental impedance data are fitted using Figure 9b.

**Figure 10:**
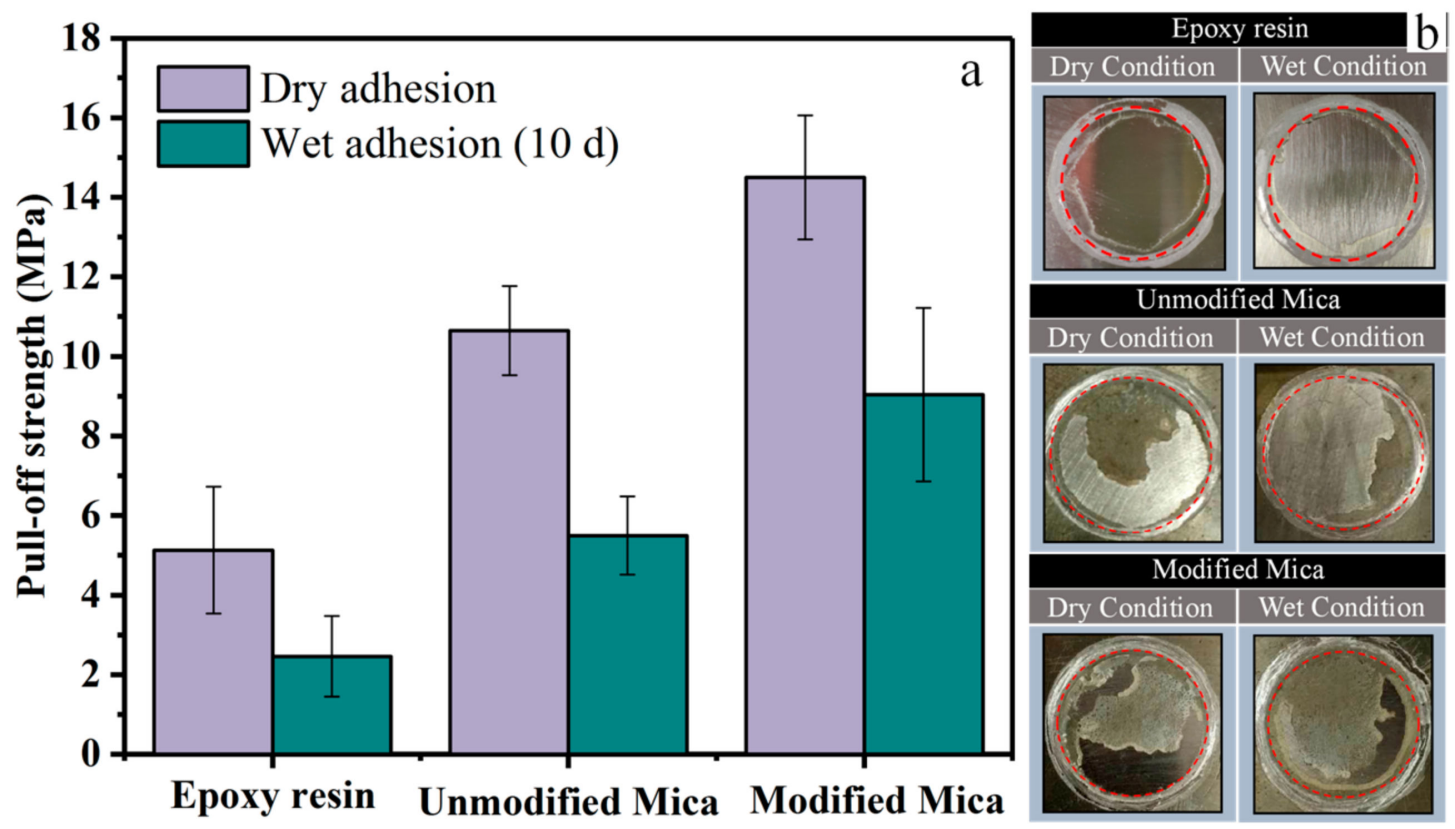
(**a**) Pull-off test results and (**b**) detaching status images.

The effect of unmodified mica and modified mica particles on the adhesion behavior of the epoxy coatings was assessed by pull-off and representative dry and wet adhesion strength, respectively. The pull-off test results, along with the detaching status images, are presented in Figure 10. The adhesion of both coatings shows a certain degree of decrease after immersion in a 3.5% NaCl solution for 10 days. However, the adhesion of the modified mica coating is much higher than that of the pure epoxy coating and unmodified mica coating in both dry and wet states. Meanwhile, the detaching status images show that the detaching areas of the pure epoxy coating and unmodified mica coating after soaking are much larger than that of the modified mica coating. Evidently, the unmodified mica particles have the capability to improve the adhesion strength, most probably due to their good compatibility with the epoxy matrix. The interface compatibility between the filler and the matrix is improved by a 10 nm epoxy layer on the surface of the modified mica particles, as shown in Figure 6. Therefore, the wet and dry adhesion of the modified mica coating is greatly improved.

**Figure 11:**
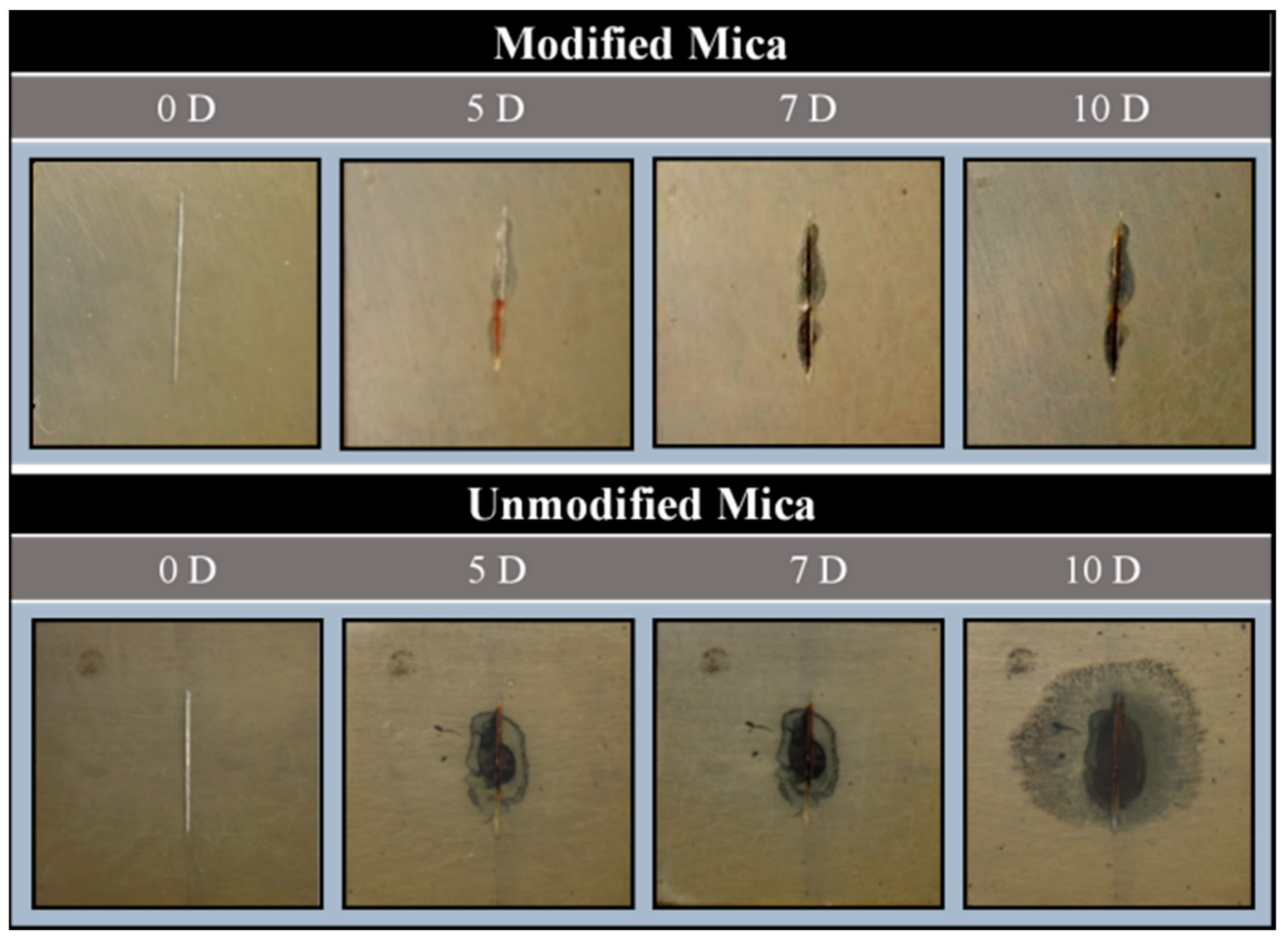
Visual performances of unmodified and modified mica coatings exposed to the salt spray test for different lengths of time.

Figure 11 shows pictures of the unmodified mica epoxy coating and modified mica epoxy coating after different durations of salt spray. There is obvious corrosion at the scratches of the two coatings, but the corrosion of the unmodified mica epoxy coating is more serious. A large number of corrosion products accumulated at the scratches of the sample, and a continuous expansion of the corrosion area can be found. At the same time, significant pitting and blistering occurred around the scratches, indicating that the coating was severely damaged. This result shows that the unmodified mica epoxy coating has a poor shielding performance against water molecules and is easily damaged by the penetration of water molecules. In contrast to the unmodified mica epoxy coating, only slight corrosion occurred in the scratches of the modified mica epoxy coating, the accumulation of corrosion products was lower, and there was less pitting and bubbling around the scratches. This finding shows that the modified mica epoxy coating significantly improves shielding performance and corrosion resistance. These improvements are mainly because of the great interface compatibility of the mica and organic epoxy, leading to a good dispersion of the two-dimensional lamellar mica in the epoxy. Therefore, the density of the coating was improved, which reduced the diffusion of water molecules into the coating.

**Figure 12:**
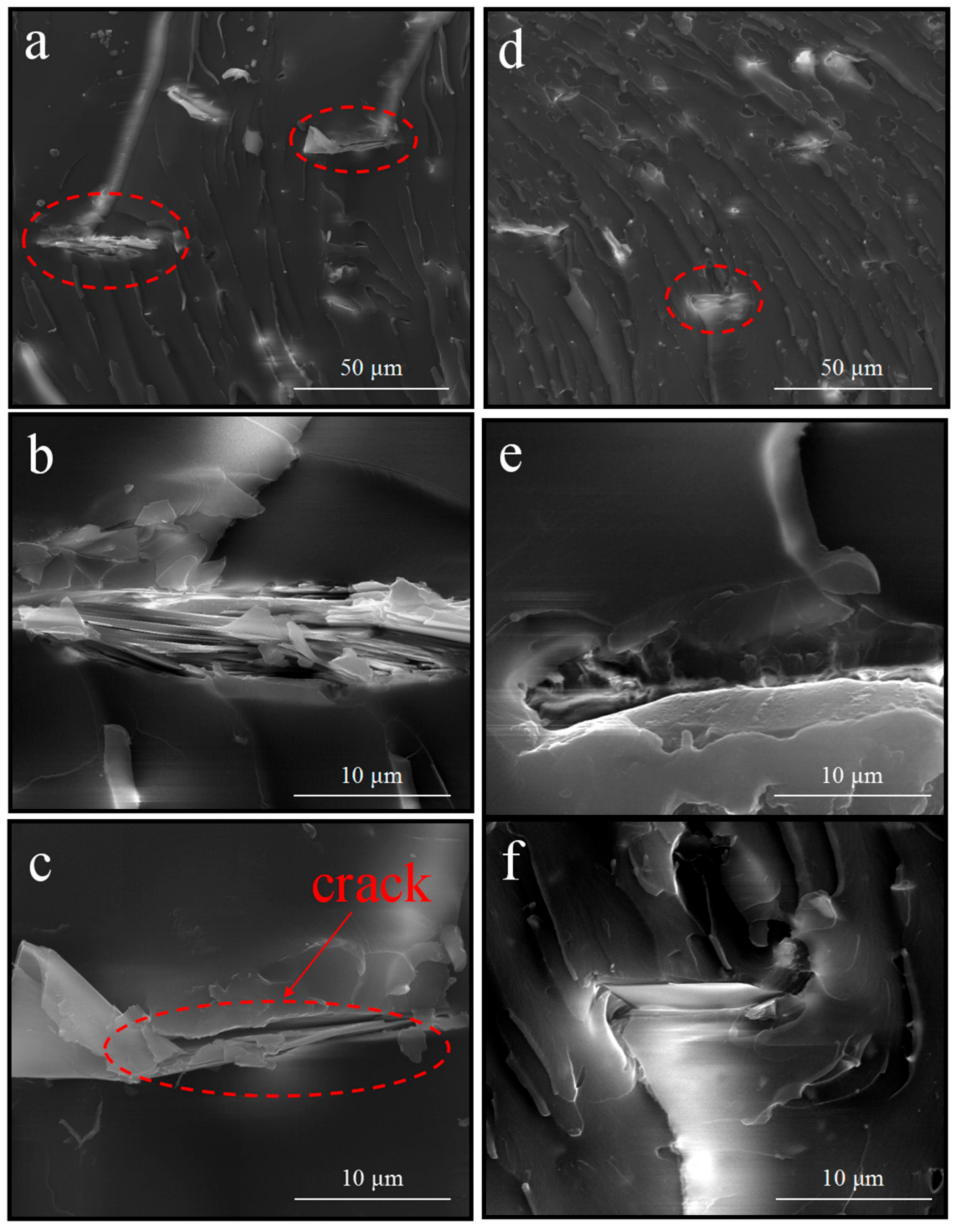
SEM images of the cross-section of (**a**–**c**) the unmodified mica coating and (**d**–**f**) the modified mica coating.

Figure 12 shows SEM images of the cross-section of (a–c) unmodified and (d–f) modified mica epoxy resin coatings, respectively. There are obvious holes in the unmodified mica epoxy coating, serious agglomeration between the particles, and obvious cracks between the agglomerated mica particles and the epoxy resin matrix, indicating that the compatibility between the mica and epoxy resin is poor. On the other hand, the pores of the modified mica epoxy coating are significantly reduced, and the mica is more evenly dispersed in the matrix, with no obvious agglomeration or cracks around the mica. According to the above analysis, the presence of the epoxy resin thin layer on the surface of mica particles improves the dispersion and interfacial compatibility of mica in the epoxy matrix and reduces the generation of pores and agglomeration. The improvement of compatibility and dispersibility is the main reason for the improvement of the comprehensive corrosion resistance of the coating.

## 4. Discussion

In this work, a high-temperature mechanochemical method was used to graft organic epoxy resin onto the surface of mica. The mechanochemical method inputting higher energy was used to modify the mica. Finally, the hydroxyl group on the surface of the mica reacted with the epoxy group in the epoxy coating, thus achieving the modification of the mica surface.

The modified mica surface formed an organic resin layer with a thickness of about 10 nm. The properties of this layer were lipophilic and hydrophobic, causing an increase in the contact angle of the mica surface from 26 to 108°, which greatly changed the mica surface properties.

The presence of the organic resin layer on the surface of the mica greatly improves the interface compatibility between the filler and the epoxy coating. The agglomeration of the filler in the coating is significantly reduced. The improvement of the coating’s compactness and toughness is the reason for its anti-corrosion performance enhancement.

## Data Availability

The data presented in this study are available on request from the corresponding author.
